# A core outcome set for pituitary surgery research: an international delphi consensus study

**DOI:** 10.1007/s11102-025-01553-w

**Published:** 2025-07-23

**Authors:** Alexandra Valetopoulou, Nicola Newall, Danyal Z. Khan, Anouk Borg, Pierre M. G. Bouloux, Fion Bremner, Michael Buchfelder, Simon Cudlip, Neil Dorward, William M. Drake, Juan C. Fernandez-Miranda, Maria Fleseriu, Mathew Geltzeiler, Joy Ginn, Mark Gurnell, Steve Harris, Zane Jaunmuktane, Márta Korbonits, Michael Kosmin, Olympia Koulouri, Hugo Layard Horsfall, Adam N. Mamelak, Richard Mannion, Pat McBride, Ann I. McCormack, Shlomo Melmed, Katherine A. Miszkiel, Gerald Raverot, Thomas Santarius, Theodore H. Schwartz, Inma Serrano, Gabriel Zada, Stephanie E. Baldeweg, Hani J. Marcus, Angelos G. Kolias, on behalf of the PitCOP Collaborators, Aaron Lawson McLean, Alasdair Mackintosh, Alexandros Boukas, Alison Julia Bryant, Andrew John Blamey, Anita J. Evans, Axel Petzold, Barry Culpin, Benedicte Decoudier, Catherine Bray, Chloe Camoccio, Christopher Allen Lindsay, Claire Briet, Claude Fabien Litre, Colin Victor Betteley, David Edward Perry, David Jonathan Collins, Deborah Hepburn, Deborah Samantha, Eimear Carolan, Dhaval Shukla, Dhruv Parikh, Eduarda Sá-Marta, Francesca Swords, Gemma Leanne Jones, Georgina Wordsworth, Ian Nigel Dibb, Jacek Kunicki, James Alexander, Jamie Lee Prochaska, Jeanette Curran, Jemma Farrell, Jenny Lindsay, Joao Paulo Almeida, John King, Jonathan Chainey, Kanna Gnanalingham, Katy Miller, Laura-Jane Evans, Maddison Broadbent, Mark Gruppetta, Martin Doughty, Martin D. Silveston, McKay Hewison, Mia Littrell, Michelle Fattorini, Mollie Pullin, Pauline Swindells, Peter Johnson Fenwick, Rachael Burnham, Ramez Wadie Kirollos, Rob Laidler, Robert Bryant, Sally-Ann Price, Shelley Jean Pomeroy, Sian Sheppard, Soham Bandyopadhyay, Sophie A. Clarke, Sunita M. C. De Sousa, Thierry Brue, Tsegazeab Laeke, Vanessa Ariza, Varun R. Kshettry

**Affiliations:** 1 National Hospital for Neurology and Neurosurgery, London, UK; 2 Hawkes Institute, Department of Computer Science, University College London, London, UK; 3 University College London Hospitals NHS Foundation Trust, London, UK; 4 University Hospital Erlangen, Erlangen, Germany; 5 Oxford University Hospitals NHS Foundation Trust, Oxford, UK; 6 Barts and The London School of Medicine, Queen Mary University of London, London, UK; 7 Stanford University School of Medicine, 213 Quarry Road, Palo Alto, USA; 8 Oregon Health & Science University, Portland, USA; 9 The Pituitary Foundation, London, UK; 10 Addenbrooke’s Hospital and University of Cambridge, Cambridge, UK; 11 Cedars-Sinai Medical Center, Los Angeles, CA USA; 12 Vincent’s Hospital Sydney, Sydney, NSW Australia; 13 Department of Endocrinology, French Reference Center for Rare Pituitary Diseases HYPO, Hospices Civils de Lyon, France; 14 New York, NY USA; 15 Keck School of Medicine, University of Southern California, Los Angeles, CA USA

**Keywords:** Pituitary surgery, Core outcome set, Delphi consensus study

## Abstract

**Purpose:**

This study aimed to develop a core outcome set (COS) for pituitary surgery to enhance the quality, efficiency and effectiveness of future pituitary adenoma surgery research.

**Methods:**

Thirty-three outcomes were identified through a systematic review of pituitary adenoma surgery outcomes and a study on patient-reported measures. These were presented in an online survey to healthcare professionals (HCPs), patients and caregivers. In the first round, participants scored each outcome’s importance on a 5-point scale (1—strongly disagree; 5—strongly agree) and could also suggest additional outcomes, which were reviewed and, if appropriate, added to existing domains. In the second round, participants re-scored the updated the list, considering group median and interquartile range scores from the previous round. Outcomes with a median score of 5 were included in the COS. A final live online consensus meeting discussed and voted on borderline outcomes (median scores 3–4).

**Results:**

The first round received 95 responses (52% HCPs, 48% patients/caregivers). Of the 33 outcomes, 16 received a median score of 5 (strongly agree), three received 4.5 and 14 received 4 (agree). Round two received 87 responses (52% HCPs, 48% patients and caregivers). Of the 33 outcomes, 14 received a median ranking of 5, two received 4.5, 15 received 4 and two received 3 (neutral). The live meeting (attended by 12 participants: 5 HCPs, 6 patients, 1 caregiver), reached consensus on the final COS, which includes 7 domains: short-term surgical outcomes; nasal outcomes; ophthalmic outcomes; endocrine outcomes; quality of life and psychological outcomes; other short-term outcomes; and disease control outcomes.

**Conclusion:**

We advocate for use of the COS in future pituitary surgery research.

**Supplementary Information:**

The online version contains supplementary material available at 10.1007/s11102-025-01553-w.

## Introduction

Pituitary adenomas (Pituitary Neuroendocrine Tumours – PitNET) are benign tumours of the pituitary gland. They are relatively common, accounting for 10–25% of intracranial tumours [1]. The primary treatment for symptomatic non-functioning pituitary adenomas (NFPA), most functioning adenomas, and asymptomatic patients with incidental tumours with anatomical characteristics requiring preventative management (e.g. chiasmal compression) is surgical resection via transsphenoidal surgery (TSS) [2–5]. Post-operatively, most patients experience favourable neurological and endocrinological recovery [6], however, recurrence occurs in approximately 30% of patients at 5 years [7]. Additionally, even in patients who achieve long-term remission, some physical and psychological symptoms persist, adversely affecting quality of life (QoL) [8].

Despite increased research activity in the field, questions regarding disease detection and optimal treatment remain unanswered. A key barrier to definitively answering such questions is the heterogeneous outcome recording in reporting of these studies. This constraint poses a challenge to the design of evidence-based treatment strategies [9]. Currently, there is no published consensus regarding which outcomes are of most utility in pituitary surgery research.

A Core Outcome Set (COS) is an ‘agreed standardised collection of outcomes which should be measured and reported, as a minimum, in all trials for a specific clinical area’. The use of COS offers several benefits. Firstly, it reduces heterogeneity across studies, facilitating quantitative evidence synthesis. Secondly, it minimise the occurrence of selective outcome reporting, ensuring a more transparent and comprehensive presentation of results. Lastly, COS plays a crucial role in identifying relevant outcomes by engaging a wide range of stakeholders, including multidisciplinary healthcare professionals, patients, carers and charity representatives [10, 11]. When developing a COS, it is vital to ensure patient-public involvement. This allows individuals with experience of the condition to be actively involved, ensuring core outcomes are relevant and aligned with their perspectives [11]. COS have been successfully developed and implemented in cauda equina syndrome, traumatic brain injury and spinal cord injury [12–14].

To improve the quality, efficiency, and impact of future pituitary surgery research, there is an urgent need to establish a consensus-based COS, which could also serve as a benchmarking tool for Pituitary Centres of Excellence. Hence, we established an international collaborative group to conduct an international Delphi consensus process, incorporating the perspective of patients, caregivers, and clinicians, to develop a COS for future pituitary adenoma surgery research. This paper forms the second part of the Pituitary Surgery Core Outcomes and Priorities (PitCOP) study. The first part identified the top 10 research priorities in pituitary adenoma surgery, helping to guide the direction of future research and resource allocation [15].

## Methods

### Study design

**Fig. 1:**
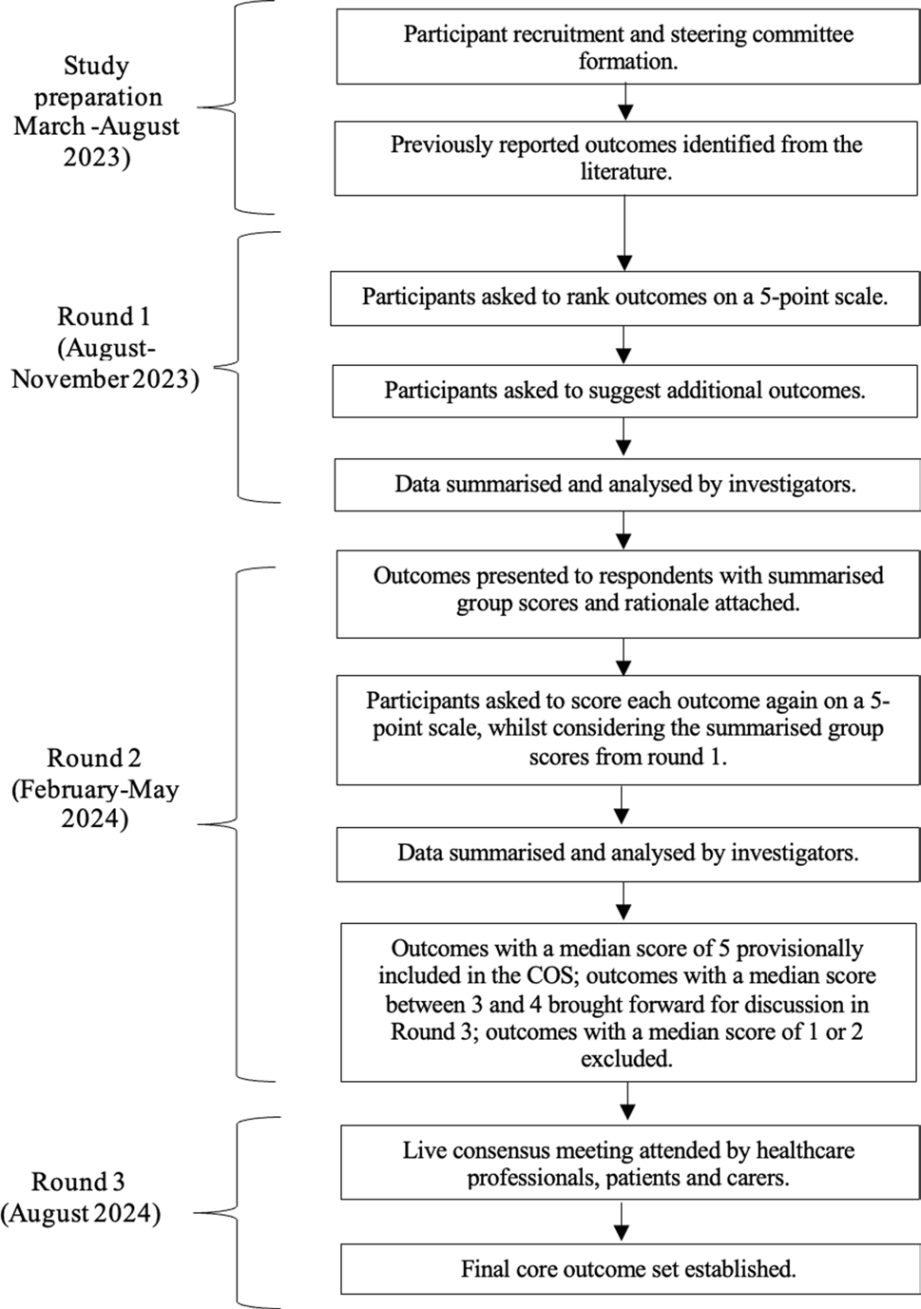
Study timeline

This study was designed and the protocol written in accordance with the Core Outcome Measures for Effectiveness Trials (COMET) Handbook, Core Outcome Set-Standards for Development (COS-STAD) and Core Outcome Set-Standardised Protocol (COS-STAP) statements [11, 16, 17]. Results were reported according to the Core Outcome Set Standards for Reporting (COS-STAR) guidelines [18]. We prospectively registered the study on the COMET database [19]. This study employed the Delphi process, a widely used and validated method for forming consensus on COS. A multi-round online Delphi survey was conducted using the online survey platform—*Qualtrics.* The study timeline is presented in Fig. 1. Ethics approval was granted by the University of Cambridge Psychology Research Ethics Committee (PRE.2023.080).

### Stakeholders and participant recruitment

Relevant stakeholder groups included: healthcare professionals (HCPs) involved in the care of patients with pituitary adenomas (including neurosurgeons, endocrinologists, ophthalmologists, oncologists, otolaryngologists, radiologists, pathologists and clinical nurse specialists); service users (including patients, their families and caregivers); and charity representatives from The Pituitary Foundation (UK charity for patients with disorders of the pituitary gland).

Promotional material was used to enhance participant recruitment and study understanding, including a dedicated study webpage and video. Invitation to participate was disseminated to relevant stakeholder groups by email and social media platforms, including the study’s official X account. The study was also promoted through the Steering Committee's professional network, as well as the relevant professional and charity organisations, including The Pituitary Foundation.

### Steering committee

A steering committee was formed to oversee study conduct and guide COS development. The committee included representatives from UK and international professional and charity organisations: The Pituitary Society, The Society of Endocrinology, The European Society of Ophthalmology and The Pituitary Foundation. The steering committee comprised 12 neurosurgeons, 10 endocrinologists, 1 ophthalmologist, 1 oncologist, 1 otolaryngologist, 1 pathologist, 1 radiologist, 1 clinical nurse specialist, 3 surgical residents, and 3 patients – who were also charity representatives of The Pituitary Foundation. The day-to-day running of the study was overseen by a management committee. Steering and management committee members are listed in supplementary Table 1.

### Phase 1 – identification of candidate outcomes

The initial list of outcomes for this study were identified from (i) a systematic review which reported outcomes after transsphenoidal surgery for pituitary adenoma, and (ii) a study which developed and validated a patient reported outcome measure (PROM) for patients with a pituitary adenoma undergoing transsphenoidal surgery [9, 20]. The list of outcomes presented in round one with their associated plain language explanations can be found in supplementary Table 2.

### Phase 2 – online surveys

#### Round 1

In the first-round, participants were presented with a list of the candidate outcomes identified during phase 1 grouped into key domains [9, 20].

Participants were then asked to rate each outcome according to how strongly they agreed or disagreed that it should be included in the COS using a 5-point scale. On the 5-point scale, 1 was ‘strongly disagree’, 2 was ‘disagree’, 3 was ‘neutral’, 4 was ‘agree’ and 5 was ‘strongly agree’. Participants were also able to provide a rationale for their rating and suggest additional outcomes for consideration. The consensus definition used throughout the study is outlined in Table 1.

**Table 1:** Consensus definition

Category	Rounds 1 and 2 (online survey)		Final consensus meeting	
	Definition	Action	Definition	Action
Consensus to include	Median score of 5 – ‘strongly agree’	After round 1: outcome included in next Delphi round. After round 2: outcome included in the final COS	As per round 1 and 2	Outcome included in final COS
Consensus to exclude	Median score of 1– ‘strongly disagree’ or 2 ‘disagree’	After round 1: outcome include in the next Delphi round. After round 2: outcomes excluded from the final COS	As per round 1 and 2	Outcome not included in final COS
No consensus	Median score of 3—‘neutral’ or 4 – ‘agree’	After round 1: outcome included in next Delphi round. After round 2: outcome brought to the consensus meeting for discussion	As per round 1 and 2	Round of discussion and voting for consensus to be reached

Data from the first round were analysed by calculating the overall median and interquartile range (IQR) for each outcome. Additional outcomes suggested by participants were reviewed by the steering (or management committee) amalgamated with existing outcomes and/or added to existing domains if deemed in scope by the steering committee. Out-of-scope outcomes were those deemed unrelated to pituitary surgery or those considered too broad to be included in the COS.

#### Round 2

Participation in the second round was restricted to those who completed the first round. Participants were presented with an updated list of outcomes (supplementary Table 3), though each outcome was accompanied by the summarised group score (median and IQR) from round 1. Participants were asked to re-score each outcome on the same 5-point scale while considering the summarised group scores from round one. To minimise attrition bias, participants were sent regular reminder emails to complete the survey.

Data from the second round were summarised and analysed by calculating the overall median and IQR for each respective outcome. Outcomes with a median score of 1 (strongly disagree) or 2 (disagree) were provisionally excluded from the final COS. Outcomes with a median score of 5 (strongly agree) were provisionally included in the final COS. Outcomes with median scores between 3 (neutral) and 4 (agree) were brought forward for further discussion in the final consensus meeting.

### Phase 3 – final consensus meeting

The final consensus meeting established the COS. Participants for this round were recruited through the Steering Committee’s network. The live online consensus meeting took place to discuss and vote which borderline outcomes (median scores between 3 and 4) should be included in the COS. Outcomes deemed for inclusion (median score 5) and exclusion (median scores 1 or 2) were also ratified during the meeting, providing an opportunity for stakeholders to express potential concerns. Throughout the meeting, participants were actively encouraged to share their perspectives and express their opinions. Prior to the meeting, participants received a comprehensive information guide, outlining the meeting's objectives, outcomes to be discussed along with their respective round two rankings, and lay explanations of each outcome.

## Results

### Online survey—round 1

Ninety-five responses were received for the first round. Respondents included 49 HCPs (52%) and 46 (48%) service users (patients and caregivers) from 14 countries (Table 2) who ranked a list of 33 outcomes on a 5-point scale. Of the 33 outcomes, 16 received an overall median ranking of 5 (strongly agree), three received an overall median ranking of 4.5 and 14 received an overall median ranking of 4 (agree). No outcomes received an overall median ranking of ≤ 3. Among the 33 outcomes, 25 received the same median ranking from both the HCP and service user groups, while the remaining outcomes differed by no more than one point on the 5-point scale (supplementary Table 4). Participants proposed 54 additional outcomes, of which 25 were deemed out-of-scope and 29 in-scope. Of the 29 in-scope outcomes, 26 were covered by other outcomes already included in the survey, while 3 were not. These 3 outcomes—need for radiotherapy, return to work/studies, and need for additional surgical intervention—were carried forward to round 2.

**Table 2:** Participant demographic information

	Round 1 (*n* = 95)	Round 2 (*n* = 87)
**Stakeholder Group**		
Healthcare Professional	**49 (52%)**	**45 (52%)**
Neurosurgeon	25	24
Endocrinologist	17	14
Other*	7	7
Service user	**46 (48%)**	**42 (48%)**
Patient	45	41
Caregiver	1	1
**Sex**		
Female	47 (49%)	40 (46%)
Male	48 (51%)	47 (54%)
**Geographical Region**		
Europe	76 (80%)	71 (82%)
North America	12 (13%)	11 (13%)
Oceania	4 (4%)	2 (2%)
Asia	2 (2%)	2 (2%)
Africa	1 (1%)	1 (1%)

^*^Other: 2 Ophthalmologists, 1 Otorhinolaryngologist, 1 Oncologist, 1 Radiologist, 1 Pathologist, 1 Clinical Nurse Specialist

### Online survey—round 2

Eighty-seven (92%) of the respondents who completed the first round, also completed the second round. This included 45 HCPs (52%) and 42 service users (48%) from 12 countries (Table 2) who ranked an updated list of 33 outcomes on the same 5-point scale, taking into consideration the summarised prior group scores for each outcome. Of the 33 outcomes, 14 received an overall median ranking of 5 (strongly agree), two received an overall median ranking of 4.5, 15 received an overall median ranking of 4 (agree) and two received an overall median ranking of 3 (neutral). No outcomes received an overall median ranking of 2 (disagree) or 1 (strongly disagree). Among the 33 outcomes, 23 received the same median ranking from both the HCP and service user groups, while the remaining outcomes differed by no more than one point on the 5-point scale (supplementary Table 5). Participants suggested 36 additional outcomes, of which 33 were deemed out-of-scope and 3 in-scope, including sexual and reproductive health issues, postoperative systemic complications, and the need for additional medical therapy. These outcomes were carried forward to a third round for further discussion and voting.

### Final consensus meeting

The online consensus meeting included 12 participants: six patients, one caregiver, two consultant neurosurgeons, two consultant endocrinologists and a consultant otorhinolaryngologist, ensuring equal representation of HCPs and patients. The meeting was facilitated by three members of the management committee (AK, AV, DZK). Outcomes with an overall median ranking of 5 (strongly agree) were reviewed but not voted on, as they were already considered part of the COS according to the consensus definition. Outcomes with a median ranking of 3 to 4 were discussed and put to an anonymous vote to determine whether they should be included in the final COS. Additional outcomes frequently suggested by participants in the second round were also discussed and put to a vote. Domain titles and outcome phrasing was also discussed and agreed upon. The final COS was subsequently established, comprising 22 clinical and patient-centred outcomes, divided into seven key domains as presented in Table 3 and supported by an accompanying video.

**Table 3:** Final core outcome set

	Domain	Outcome
1	**Short-term surgical outcomes**	Intraoperative arterial injury
		Post-operative cerebrospinal fluid leak
		Infection (including meningitis)
2	**Nasal outcomes**	Reduced/absent sense of smell/taste
3	**Ophthalmic outcomes**	Visual acuity improvement/deterioration
		Visual fields improvement/deterioration
4	**Endocrine outcomes**	New hypopituitarism post-operatively
		Recovery of pituitary function post-operatively
		Post-operative dysnatraemia
		Sexual and reproductive health issues
5	**Quality of life and psychological outcomes**	Impact on usual activities of daily living
		Impact on mental health
6	**Other short-term outcomes**	Post-operative systemic complications (within 30 days)
		Re-operation (within 30 days)
		Re-admission (within 30 days)
		Death
7	**Disease control**	Extent of resection
		Remission (functioning adenomas)
		Recurrent disease (biochemical or radiological)
		Need for additional medical therapy
		Need for radiotherapy
		Re-operation (after 30 days)

## Discussion

This study has established a COS for use in future pituitary surgery research. An international modified Delphi process was used to gain consensus among key stakeholders regarding the most important outcomes to be included in the COS. The final COS includes a comprehensive list of 22 clinical and patient-centred outcomes, divided into seven key domains. It is recommended that future pituitary surgery research includes measures of these outcomes as a minimum. To our knowledge, this is the first attempt at developing a COS for pituitary surgery research.

### Principal findings

The seven COS domains include: short-term surgical outcomes; nasal outcomes; ophthalmic outcomes; endocrine outcomes; quality of life and psychological outcomes; other short-term outcomes; and disease control outcomes.

While TSS for pituitary adenoma resection has undergone significant technological advancements in recent years, numerous challenges remain [21]. For example, achieving safe resection of large sellar masses invading the cavernous sinus while preserving critical neurovascular structures is difficult, often impacting the extent of resection and overall surgical outcomes [21]. Therefore, documenting surgical outcomes such as intra-operative arterial injury as well as peri-operative mortality rate—even though rare (≤ 1%), is essential, particularly as new surgical techniques are introduced into clinical practice [22, 23]. Post-operative cerebrospinal fluid (CSF) leak after TSS is a well-recognised complication, occurring in up to 5% of patients. A CSF leak has potentially serious sequelae, including meningitis, prolonged hospital stay, re-admission and need for a further operation [24, 25]. Recent innovations in surgical technology and the increasing use of artificial intelligence (AI) offer promising solutions. For example, neural networks may effectively predict CSF rhinorrhoea as well as identify key predictors [26]. With more studies assessing these outcomes, more data will become available, which could further refine predictive models to enhance risk stratification, support surgical decision-making, optimise post-operative management, and enable personalised patient counselling [26]. AI-driven early-warning systems for complications have the potential to transform post-operative pituitary surgery care. For example, high-risk patients may benefit from closer monitoring and targeted preventive measures, while low-risk patients could be considered for earlier discharge, potentially reducing hospital-acquired infection risks [27, 28].

A significant proportion of patients experience post-operative olfactory dysfunction, which is more commonly transient but is sometimes permanent, having a profound impact on patients’ QoL [29, 30]. Olfactory dysfunction can result in anxiety, depression and social isolation, reduced appetite and an inability to detect dangerous odour signals [31–33]. Beyond the outcome of ‘reduced sense of smell/taste’, as incorporated in the COS other important nasal outcomes should be considered. For example, the Sinonasal Outcomes Test (SNOT) is a comprehensive tool used to evaluate sinonasal morbidity, which includes additional outcomes such as nasal congestion, discharge, impaired sleep function. Studies have demonstrated it is an excellent method for assessing sinonasal quality of life by comparing pre- and post-operative results [34]. The existing evidence on olfactory dysfunction is heterogeneous and of low-level [35]. Including nasal outcomes in the COS will help ensure that post-operative olfactory dysfunction is systematically reported, monitored for and managed in future studies.

While visual deficits usually improve after pituitary surgery, a meta-analysis found that complete recovery is achieved in only 30–40% of patients, with post-operative visual deterioration occurring in up to 4% [36]. Including ophthalmic outcomes in future studies is essential for gaining a better understanding of the factors that may contribute to incomplete recovery or deterioration. Studies should systematically report formal assessments of visual acuity and fields both pre-operatively and post-operatively, essential for accurately monitoring changes in visual function [36, 37].

Endocrine outcomes are central to pituitary surgery, particularly regarding hypopituitarism recovery and management of post-operative electrolyte imbalances. Post-operative hyponatraemia occurs in 9–39% of patients, and while asymptomatic for some patients, others may require intensive care admission—increasing the morbidity associated with pituitary surgery [38]. Pituitary surgery induces new hypopituitarism in < 10% of patients, while 30% show improved pituitary function post-operatively [39]. In patients with Cushing’s disease, the risk of post-operative hypopituitarism is increased by up to 50%, particularly following repeat surgeries [40]. Additionally, the reported incidence of post-operative Arginine Vasopressin Deficiency (AVP-D), previously named Diabetes Insipidus, can be as high as 30% and approximately 2% for permanent AVP-D [39, 41]. Given its prevalence as a postoperative complication, accurately predicting transient AVP-D is crucial [42]. Incorporating such outcomes into future studies will generate more comprehensive information, facilitating comprehensive exploration of clinical factors. This, in turn, will enable HCPs to implement targeted preventive measures and personalized treatment strategies [43, 44]. The emerging use of AI also offers promising solutions such as predicting post-operative AVP-D using machine learning algorithms, demonstrating that shorter pituitary stalk, and lower pre-operative ACTH and cortisol levels were associated with a higher probability of developing AVP-D post-operatively [45].

The importance of understanding and evaluating the impact of pituitary surgery on patients’ mental wellbeing and QoL is critical yet often overlooked. Most studies have primarily focused on objective clinical outcomes, neglecting the psychological effects that pituitary surgery has on patients and their carers [46]. This gap was particularly evident in the first two Delphi rounds of the study, where patients consistently highlighted the psychological challenges they experienced and the lack of resources and support available. Patients harbouring pituitary adenomas experience psychological distress, with studies demonstrating that their QoL may not improve even after a clinically successful operation [47]. Accordingly, sexual and reproductive health symptoms—an often-underreported aspect of pituitary surgery [48]—have been incorporated into the COS. Participants frequently cited infertility, reduced libido, and sexual dysfunction, highlighting the significant impact these issues have on overall well-being and QoL. Patient-reported outcome measures (PROMs) have been tailored specifically for individuals undergoing pituitary adenoma surgery, which guided our initial list of outcomes [20]. Including QoL and psychological outcomes in the COS, as well as promoting the use and validation of PROMs, will help ensure a more comprehensive evaluation of postoperative patient wellbeing. This will facilitate a deeper understanding of the complex psychosocial challenges faced by patients, families, and caregivers throughout the treatment journey, ultimately aligning future research more closely with their perspectives.

TSS has a relatively low 30-day re-operation rate, with the most common indications being CSF leak repair, control of epistaxis and post-operative haematoma evacuation [49–53]. Limited data exists on the risk factors and outcomes associated with the need for re-operation for evacuation of post-operative hematoma [51]. Consequently, clinical and tumour characteristics which could predict post-operative hematoma requiring re-operation remain poorly defined. Identifying these predictors could enable stratification of high-risk patients who may benefit from closer post-operative monitoring and provide valuable outcome data to assist clinicians in pre-operative planning [54]. Re-admission within 30 days post TSS is relatively rare, with reported rates ranging from 6 to 9% and delayed hyponatremia being the leading cause [55, 56]. Recording re-admission rates and indications is a valuable key quality-of-care metric and helps identify high-risk patient populations [57, 58]. Patients with hypertension, hypothyroidism, and diabetes have a higher risk of post-operative systemic complications and re-admission [59–61]. However, there is limited data on 30-day re-admission rates after TSS, and predictive factors have not been clearly identified. Determining clinically relevant predictors of re-admission and understanding the typical timeframe of complications after TSS could optimise inpatient management, discharge planning, and post-operative follow-up plans, aiming to reduce preventable re-admissions [62, 63].

Recurrence for NFPAs remains high despite advancements in surgical techniques, with rates between 15–60% for those treated with surgery alone and 2–28% in patients treated with surgery and radiotherapy [64–66]. Additionally, many series report high rates of recurrence in functioning adenomas, such as up to 20% in Cushing’s disease [67]. Comprehensive research on clinical factors affecting recurrence rates remains limited, emphasising the need for future studies to focus on identifying prognostic factors [68]. Furthermore, the consensus process also emphasised the need for more data on additional medical therapy post-operatively. For example, while existing evidence indicates that dopamine agonists may benefit NFPA patients with residual tumour, the overall evidence remains limited [64, 69]. Additionally, remission of functioning adenomas is a key treatment goal but remains challenging to define due to the limitations of current diagnostic criteria and variability in individual responses to surgical and adjuvant therapies [70, 71]. This outcome is particularly valuable in Cushing’s disease, as it informs clinical decision-making, including whether to pursue a more aggressive surgical resection [72, 73]. Although there is demonstrated potential of machine learning (ML) algorithms in identifying risk factors and predicting recurrence and remission [74–78], further research is required to develop and optimise high-performing ML models for identifying clinically relevant factors. Incorporating recurrence and remission rates in future pituitary surgery studies will generate valuable data, facilitating the development of prediction models to assess treatment response and forecast recurrence risk. These innovations could improve post-operative risk assessment precision and optimise long-term management strategies.

### Strengths and limitations

This study has several strengths. First, it was rigorously designed in accordance with the COMET Handbook and adhered to all established COS development and reporting guidelines [11, 16–18]. A key strength of the study is the use of the Delphi process, a well-established and validated method for gaining consensus [11]. This facilitated meaningful input from a diverse range of stakeholders, including HCPs, patients, caregivers and charity representatives. Furthermore, there was equal representation of both HCPs and patients across all three rounds of the consensus process. Active involvement of patients and caregivers is critical in ensuring their perspectives are integrated into the COS, reducing the risk of overlooking important patient-centred outcomes. Strong participant engagement and commitment was evident throughout the study. In the second round, restricted to those who completed the prior round, 87 of 95 participants (92%) continued their involvement. Additionally, user-friendly promotional materials; posters, animated videos, presentations and articles, further enhanced participant recruitment and raise study awareness. These materials were developed with guidance from public engagement specialists, ensuring that the study’s aims were effectively communicated, facilitating broad participation.

There are, however, limitations which need to be acknowledged. Despite the efforts made for international dissemination, there was underrepresentation of participants from low- and middle-income countries. Although the study had global reach, most participants were from Europe, limiting the diversity of experiences drawn from different healthcare and research systems. This lack of geographical representation can impact the comprehensiveness of the outcomes captured, as it may not fully reflect the unique challenges and perspectives of patients and healthcare providers in other settings.

Additionally, as the first two rounds of the study were conducted online, there were potential accessibility issues for individuals with limited technical literacy or visual impairment. While online surveys are efficient and accessible to many, they may unintentionally exclude certain populations.

### Implications and future research

It is important to emphasise that outcomes included in a COS are the *minimum* outcomes that should be assessed in future research studies. However, researchers are strongly encouraged to incorporate and assess other outcomes in addition to the COS wherever possible. For example, optical coherence tomography (OCT) metrics, were included in our initial outcome list, but were excluded from the final COS, as OCT is not routinely used/readily accessible in some centres. However, where relevant, OCT metrics may be a useful outcome to record given evidence suggesting its value as a prognostic tool for post-operative visual function outcomes in some patients [79].

While the COS has defined ‘what’ to report, it is also important to establish ‘how’ these outcomes should be measured. Therefore, further work is needed to identify the best instruments and define ways to measure individual outcomes included in the COS – known as a core measurement set [80]. Additionally, next steps should include development of core data elements—a set of essential characteristics that need to be reported to facilitate the interpretation of the core outcomes.

Furthermore, given the potential bias introduced by the underrepresentation of participants from low- and middle-income countries, it would be beneficial to validate the COS in different global regions. Finally, successful implementation will require ongoing and collaborative efforts, with relevant societies adopting these outcomes. This will ensure collection of sufficient data to enable comparisons and evidence synthesis in systematic reviews and meta-analyses. Such efforts will facilitate consistent and rigorous evaluation of key outcomes, ultimately leading to meaningful conclusions that can inform the adoption of novel treatments/interventions and highlight gaps in research. It will be important to evaluate the uptake and use of this COS in standardising selection and reporting of outcomes across pituitary surgery research. This will be tracked by identifying how frequently the COS is cited as well as by conducting systematic reviews – identifying research studies which have employed it. The published COS will be shared on the study’s webpage and social media platforms as well as relevant professional and charity organisations to inform and guide pituitary surgery researchers.

## Supplementary Information

Below is the link to the electronic supplementary material.

Supplementary file1 (DOCX 19 KB)

Supplementary file2 (DOCX 18 KB)

Supplementary file3 (DOCX 19 KB)

Supplementary file4 (DOCX 21 KB)

Supplementary file5 (DOCX 21 KB)

## Data Availability

No datasets were generated or analysed during the current study.
