# Supplementary material for: A core outcome set for pituitary surgery research: an international delphi consensus study
Source: Pituitary. 2025 Jul 23;28(4):88. doi: 10.1007/s11102-025-01553-w (PMC12287136; doi:10.1007/s11102-025-01553-w)
Supplement: Supplementary file 1 — Supplementary file1 (DOCX 19 KB) [file 11102_2025_1553_MOESM1_ESM.docx]

**Journal: Pituitary**

**Title: A Core Outcome Set for Pituitary Surgery Research: An International Delphi Consensus Study**

Alexandra Valetopoulou^1,2^, Nicola Newall^1,2^, Danyal Z Khan^1,2^, Anouk Borg^1^ , Pierre M G Bouloux^3^, Fion Bremner^1^, Michael Buchfelder^4^, Simon Cudlip^5^, Neil Dorward^1^, William M Drake^6^, Juan C Fernandez-Miranda^7^, Maria Fleseriu^8^, Mathew Geltzeiler^8^, Joy Ginn^9^, Mark Gurnell^10^, Steve Harris^9^, Zane Jaunmuktane^1^, Márta Korbonits^6^, Michael Kosmin^3^, Olympia Koulouri^10^, Hugo Layard Horsfall^1,2^, Adam N Mamelak^11^ ,Richard Mannion^10^, Pat McBride^9^, Ann I McCormack^12^, Shlomo Melmed^11^,Katherine A Miszkiel^1^, Gerald Raverot^13^, Thomas Santarius^10^, Theodore H Schwartz^14^, Inma Serrano^1^, Gabriel Zada^15^, *Stephanie E Baldeweg^3^,*Hani J Marcus^1,2^,*Angelos G Kolias^10^, on behalf of the PitCOP Collaborators**

^1^National Hospital for Neurology and Neurosurgery, London, United Kingdom

^2^Hawkes Institute, Department of Computer Science, University College London, United

Kingdom

^3^University College London Hospitals NHS Foundation Trust, London, United Kingdom

^4^University Hospital Erlangen, Erlangen, Germany

^5^Oxford University Hospitals NHS Foundation Trust, Oxford, United Kingdom

^6^Barts and The London School of Medicine, Queen Mary University of London, London, United Kingdom

^7^Stanford University School of Medicine, 213 Quarry Road, Palo Alto, USA

^8^Oregon Health & Science University, Portland, USA

^9^The Pituitary Foundation, United Kingdom

^10^Addenbrooke’s Hospital and University of Cambridge, Cambridge, UK

^11^Cedars-Sinai Medical Center, Los Angeles, CA, United States

^12^St Vincent's Hospital Sydney, Sydney, NSW, Australia

^13^Department of Endocrinology, French Reference Center for Rare Pituitary Diseases HYPO, Hospices Civils de Lyon, France

^14^Weill Cornell Medical College, New York, NY, USA

^15^Keck School of Medicine, University of Southern California, Los Angeles, CA, USA.

*Joint senior authors.

** PitCOP Collaborators are listed in the Acknowledgments section.

Corresponding Author: Alexandra Valetopoulou

Corresponding Author’s email address: [alexandra.valetopoulou@gmail.com](mailto:alexandra.valetopoulou@gmail.com)

**Supplementary information 1a: Steering committee members**

| Steering Committee Member | Stakeholder group |
| --- | --- |
| Adam Mamelak | Neurosurgery |
| Alexandra Valetopoulou | Neurosurgery Resident |
| Angelos Kolias | Neurosurgery |
| Ann McCormack | Endocrinology |
| Anouk Borg | Neurosurgery |
| Danyal Z Khan | Neurosurgery Resident |
| Fion Bremner | Ophthalmology |
| Gabriel Zada | Neurosurgery |
| Gerald Raverot | Endocrinology |
| Hani Marcus | Neurosurgery |
| Inma Serrano | Clinical Nurse Specialist |
| Joy Ginn | Patient and Charity Representative |
| Juan Fernandez-Miranda | Neurosurgery |
| Katherine Miszkiel | Radiology |
| Maria Fleseriu | Endocrinology |
| Mark Gurnell | Endocrinology |
| Márta Korbonits | Endocrinology |
| Mathew Geltzeiler | Otolaryngology |
| Michael Buchfelder | Neurosurgery |
| Michael Kosmin | Oncology |
| Neil Dorward | Neurosurgery |
| Nicola Newall | Neurosurgery Resident |
| Olympia Koulouri | Endocrinology |
| Pat McBride | Patient and Charity Representative |
| Pierre Bouloux | Endocrinology |
| Richard Mannion | Neurosurgery |
| Shlomo Melmed | Endocrinology |
| Simon Cudlip | Neurosurgery |
| Stephanie Baldeweg | Endocrinology |
| Steve Harris | Patient and Charity Representative |
| Theodore Schwartz | Neurosurgery |
| Tom Santarius | Neurosurgery |
| William Drake | Endocrinology |
| Zane Jaunmuktane | Pathology |

**Supplementary information 1b: Management committee members**

| Management Committee Member | Stakeholder group |
| --- | --- |
| Hani Marcus | Neurosurgery |
| Angelos Kolias | Neurosurgery |
| Stephanie Baldeweg | Endocrinology |
| Danyal Z Khan | Neurosurgery Resident |
| Nicola Newall | Neurosurgery Resident |
| Alexandra Valetopoulou | Neurosurgery Resident |
