# Supplementary material for: A core outcome set for pituitary surgery research: an international delphi consensus study
Source: Pituitary. 2025 Jul 23;28(4):88. doi: 10.1007/s11102-025-01553-w (PMC12287136; doi:10.1007/s11102-025-01553-w)
Supplement: Supplementary file 2 — Supplementary file2 (DOCX 18 KB) [file 11102_2025_1553_MOESM2_ESM.docx]

**Journal: Pituitary**

**Title: A Core Outcome Set for Pituitary Surgery Research: An International Delphi Consensus Study**

Alexandra Valetopoulou^1,2^, Nicola Newall^1,2^, Danyal Z Khan^1,2^, Anouk Borg^1^ , Pierre M G Bouloux^3^, Fion Bremner^1^, Michael Buchfelder^4^, Simon Cudlip^5^, Neil Dorward^1^, William M Drake^6^, Juan C Fernandez-Miranda^7^, Maria Fleseriu^8^, Mathew Geltzeiler^8^, Joy Ginn^9^, Mark Gurnell^10^, Steve Harris^9^, Zane Jaunmuktane^1^, Márta Korbonits^6^, Michael Kosmin^3^, Olympia Koulouri^10^, Hugo Layard Horsfall^1,2^, Adam N Mamelak^11^ ,Richard Mannion^10^, Pat McBride^9^, Ann I McCormack^12^, Shlomo Melmed^11^,Katherine A Miszkiel^1^, Gerald Raverot^13^, Thomas Santarius^10^, Theodore H Schwartz^14^, Inma Serrano^1^, Gabriel Zada^15^, *Stephanie E Baldeweg^3^,*Hani J Marcus^1,2^,*Angelos G Kolias^10^, on behalf of the PitCOP Collaborators**

^1^National Hospital for Neurology and Neurosurgery, London, United Kingdom

^2^Hawkes Institute, Department of Computer Science, University College London, United

Kingdom

^3^University College London Hospitals NHS Foundation Trust, London, United Kingdom

^4^University Hospital Erlangen, Erlangen, Germany

^5^Oxford University Hospitals NHS Foundation Trust, Oxford, United Kingdom

^6^Barts and The London School of Medicine, Queen Mary University of London, London, United Kingdom

^7^Stanford University School of Medicine, 213 Quarry Road, Palo Alto, USA

^8^Oregon Health & Science University, Portland, USA

^9^The Pituitary Foundation, United Kingdom

^10^Addenbrooke’s Hospital and University of Cambridge, Cambridge, UK

^11^Cedars-Sinai Medical Center, Los Angeles, CA, United States

^12^St Vincent's Hospital Sydney, Sydney, NSW, Australia

^13^Department of Endocrinology, French Reference Center for Rare Pituitary Diseases HYPO, Hospices Civils de Lyon, France

^14^Weill Cornell Medical College, New York, NY, USA

^15^Keck School of Medicine, University of Southern California, Los Angeles, CA, USA.

*Joint senior authors.

** PitCOP Collaborators are listed in the Acknowledgments section.

Corresponding Author: Alexandra Valetopoulou

Corresponding Author’s email address: [alexandra.valetopoulou@gmail.com](mailto:alexandra.valetopoulou@gmail.com)

**Supplementary Information 2: Round one outcomes with their associated plain language summary**

**Domain 1: Surgical outcomes**

**Intraoperative arterial injury**During the operation, there is a small risk of damaging important blood vessels/arteries of the brain such as the carotid artery. Damage to these arteries may cause bleeding into the brain or stroke.

**Post-operative cerebrospinal fluid leak**Cerebrospinal fluid is the fluid that surrounds the brain, this may leak from the nose after surgery. In some cases, another surgery might be required to repair the leak.

**Infection (including meningitis)**Meningitis is an infection affecting the lining of the brain.

**Epistaxis requiring intervention**Epistaxis is the medical term for a nosebleed, this may occur if a blood vessel is damaged during surgery.

**Resection cavity haematoma**Collection of blood in the operative cavity after the operation. In some cases this might require a second operation to remove the blood.

**Need for reoperation (including the indication)**This refers to the need for a second operation and the reason why this may be required.

**Extent of resection**This refers to the proportion of the tumour that has been successfully removed.

**Recurrent disease**This refers to when a pituitary tumour which was thought to have been fully removed during the operation regrows.

**Domain 2: Nasal outcomes**

**Nasal congestion**This is the medical term for experiencing a feeling of fullness in the nose.

**Nasal discharge**This is the medical term for a runny nose.

**Anosmia**This is the medical term for loss of smell.

**Domain 3: Ophthalmic outcomes**

**Visual acuity improvement/deterioration**Visual acuity refers to the clarity of vision.

**Visual fields improvement/deterioration**

This refers to the total area in which objects can be seen in your peripheral vision when you focus your eyes on a central point.

**Colour vision improvement/deterioration**

This refers to the ability of the eyes to distinguish different colours.

**Diplopia**This is the medical term for double vision.

**Optic disc grading**The optic disc, sometimes called the optic nerve head, is a round section at the back of the eye ball. This area is often damaged in patients with pituitary tumours affecting their vision. This damage can sometimes be improved or reversed after the operation.

**Optical coherence tomography (OCT) metrics**

Optical Coherence Tomography (OCT) metrics refer to the various measurements and data obtained from an OCT scan. OCT is a non-invasive imaging test that uses light waves to take detailed pictures of the inside of the eye.

**Domain 4: Endocrine outcomes**

**Transient/Permanent hypopituitarism**The pituitary gland is responsible for producing hormones and chemicals which control other glands in our body. If the pituitary is not producing one or more of these hormones, or not producing enough, then this is known as hypopituitarism. 'Transient' hypopituitarism refers to this being temporary and reversible. 'Permanent' hypopituitarism refers to this being irreversible.

**Transient/Permanent diabetes insipidus**Diabetes insipidus (DI) is caused by a problem with either the production, or action, of a hormone which controls your kidneys’ ability to retain water in the body. If you have DI - your kidneys are unable to retain water, resulting in the production of too much urine. 'Transient' DI refers to this being temporary and reversible. 'Permanent' DI refers to this being irreversible.

**Syndrome of inappropriate antidiuretic hormone secretion**Syndrome of inappropriate antidiuretic hormone (SIADH) secretion is a condition where the pituitary gland produces too much antidiuretic hormone (ADH). This hormone control the amount of water the body loses in urine. SIADH causes the body to retain too much water.

**Remission (functioning adenomas)**Reduction or disappearance of the signs and symptoms caused by pituitary tumours which produce hormones.

**Domain 6: Other short-term outcomes**

**Length of hospital stay**This refers to the number of days a patient is admitted to hospital for.

**Readmission (within 30 days of operation, including the indication)**If a further hospital admission is required within 30 days of the initial operation including the reason for the second admission.
