# Supplementary material for: A core outcome set for pituitary surgery research: an international delphi consensus study
Source: Pituitary. 2025 Jul 23;28(4):88. doi: 10.1007/s11102-025-01553-w (PMC12287136; doi:10.1007/s11102-025-01553-w)
Supplement: Supplementary file 5 — Supplementary file5 (DOCX 21 KB) [file 11102_2025_1553_MOESM5_ESM.docx]

**Journal: Pituitary**

**Title: A Core Outcome Set for Pituitary Surgery Research: An International Delphi Consensus Study**

Alexandra Valetopoulou^1,2^, Nicola Newall^1,2^, Danyal Z Khan^1,2^, Anouk Borg^1^ , Pierre M G Bouloux^3^, Fion Bremner^1^, Michael Buchfelder^4^, Simon Cudlip^5^, Neil Dorward^1^, William M Drake^6^, Juan C Fernandez-Miranda^7^, Maria Fleseriu^8^, Mathew Geltzeiler^8^, Joy Ginn^9^, Mark Gurnell^10^, Steve Harris^9^, Zane Jaunmuktane^1^, Márta Korbonits^6^, Michael Kosmin^3^, Olympia Koulouri^10^, Hugo Layard Horsfall^1,2^, Adam N Mamelak^11^ ,Richard Mannion^10^, Pat McBride^9^, Ann I McCormack^12^, Shlomo Melmed^11^,Katherine A Miszkiel^1^, Gerald Raverot^13^, Thomas Santarius^10^, Theodore H Schwartz^14^, Inma Serrano^1^, Gabriel Zada^15^, *Stephanie E Baldeweg^3^,*Hani J Marcus^1,2^,*Angelos G Kolias^10^, on behalf of the PitCOP Collaborators**

^1^National Hospital for Neurology and Neurosurgery, London, United Kingdom

^2^Hawkes Institute, Department of Computer Science, University College London, United

Kingdom

^3^University College London Hospitals NHS Foundation Trust, London, United Kingdom

^4^University Hospital Erlangen, Erlangen, Germany

^5^Oxford University Hospitals NHS Foundation Trust, Oxford, United Kingdom

^6^Barts and The London School of Medicine, Queen Mary University of London, London, United Kingdom

^7^Stanford University School of Medicine, 213 Quarry Road, Palo Alto, USA

^8^Oregon Health & Science University, Portland, USA

^9^The Pituitary Foundation, United Kingdom

^10^Addenbrooke’s Hospital and University of Cambridge, Cambridge, UK

^11^Cedars-Sinai Medical Center, Los Angeles, CA, United States

^12^St Vincent's Hospital Sydney, Sydney, NSW, Australia

^13^Department of Endocrinology, French Reference Center for Rare Pituitary Diseases HYPO, Hospices Civils de Lyon, France

^14^Weill Cornell Medical College, New York, NY, USA

^15^Keck School of Medicine, University of Southern California, Los Angeles, CA, USA.

*Joint senior authors.

** PitCOP Collaborators are listed in the Acknowledgments section.

Corresponding Author: Alexandra Valetopoulou

Corresponding Author’s email address: [alexandra.valetopoulou@gmail.com](mailto:alexandra.valetopoulou@gmail.com)

**Supplementary Information 5: Round two outcome ratings**

|  | **Domain** | **Outcome** | **Service users (n=42)** | **HCPs (n=45)** | **Overall (n=87)** |
| --- | --- | --- | --- | --- | --- |
|  |  |  | **Median (IQR)** | **Median (IQR)** | **Median (IQR)** |
| **1** | **Surgical outcomes** | Intraoperative arterial injury | 5 (3.25-5) | 5 (5-5) | 5 (4-5) |
|  |  | Post-operative cerebrospinal fluid leak | 5 (4-5) | 5 (5-5) | 5 (4-5) |
|  |  | Infection (including meningitis) | 5 (4-5) | 5 (4-5) | 5 (4-5) |
|  |  | Epistaxis requiring intervention | 4 (3-4) | 4 (4-5) | 4 (3-4.75) |
|  |  | Resection cavity haematoma requiring intervention | 4 (3-5) | 5 (4-5) | 4 (3.25-5) |
|  |  | Extent of resection | 4 (3.25-5) | 5 (4-5) | 5 (4-5) |
|  |  | Recurrent disease | 5 (4-5) | 5 (4-5) | 5 (4-5) |
|  |  | Death | 5 (4-5) | 5 (5-5) | 5 (4.25-5) |
|  |  | Need for additional surgical intervention (e.g. lumbar drain) | 4.5 (4-5) | 4.5 (4-5) | 4.5 (4-5) |
| **2** | **Nasal outcomes** | Nasal congestion | 4 (3-5) | 4 (3-4) | 4 (3-4) |
|  |  | Nasal discharge | 4 (3-5) | 3.5 (3-4) | 4 (3-4) |
|  |  | Altered sense of smell/taste | 4 (4-5) | 4 (4-5) | 4 (4-5) |
|  |  | Anosmia | 4 (3.25-5) | 4 (4-5) | 4 (4-5) |
|  |  | Headaches | 5 (4-5) | 4 (3-4) | 4 (3-5) |
| **3** | **Ophthalmic outcomes** | Visual acuity improvement/deterioration | 5 (4-5) | 5 (4-5) | 5 (4-5) |
|  |  | Visual fields improvement/deterioration | 5 (4-5) | 5 (5-5) | 5 (4-5) |
|  |  | Colour vision improvement/deterioration | 4 (3-5) | 4 (3-4) | 4 (3-4) |
|  |  | Diplopia | 4 (4-5) | 5 (4-5) | 4 (4-5) |
|  |  | Optic disc grading | 4 (3-4) | 3 (3-4) | 3 (3-4) |
|  |  | Optical coherence tomography (OCT) metrics | 3 (3-4) | 3 (3-4) | 3 (3-4) |
| **4** | **Endocrine outcomes** | New hypopituitarism following surgery | 5 (4-5) | 5 (4-5) | 5 (4-5) |
|  |  | Recovery of pituitary function post-operatively | 5 (4-5) | 4.5 (4-5) | 5 (4-5) |
|  |  | Post-operative dysnatraemia | 4 (4-5) | 4 (3.75-5) | 4 (4-5) |
|  |  | Remission (functioning adenomas) | 5 (4-5) | 5 (5-5) | 5 (5-5) |
| **5** | **Quality of life and psychological outcomes** | Impact on usual activities of daily living | 5 (5-5) | 4 (4-5) | 5 (4-5) |
|  |  | Impact on mobility | 4 (4-5) | 4 (3-4) | 4 (3-5) |
|  |  | Pain or Discomfort | 5 (4-5) | 4 (3.75-4) | 4 (4-5) |
|  |  | Impact on mental health | 5 (4-5) | 4 (3-4) | 4 (3-5) |
|  |  | Return to work/studies | 5 (4-5) | 4 (4-5) | 4 (4-5) |
| **6** | **Other short-term outcomes** | Length of hospital stay | 4 (3-4.75) | 4 (3.75-5) | 4 (3-5) |
|  |  | Re-admission (within 30 days of operation, including the indication) | 4 (3-5) | 5 (4-5) | 5 (4-5) |
| **7** | **Need for additional treatment** | Need for re-operation (including the indication e.g. residual, re-growth) | 5 (4-5) | 5 (4-5) | 5 (4-5) |
|  |  | Need for radiotherapy | 5 (4-5) | 4 (4-5) | 4.5 (4-5) |
